# Mobile Network Data for Public Health: Opportunities and Challenges

**DOI:** 10.3389/fpubh.2015.00189

**Published:** 2015-08-07

**Authors:** Nuria Oliver, Aleksandar Matic, Enrique Frias-Martinez

**Affiliations:** ^1^Telefónica Research, Barcelona, Spain

**Keywords:** public health, cell phone, mobile data, CDR

## Abstract

The ubiquity of mobile phones worldwide is generating an unprecedented amount of human behavioral data both at an individual and aggregated levels. The study of this data as a rich source of information about human behavior emerged almost a decade ago. Since then, it has grown into a fertile area of research named *computational social sciences* with a wide variety of applications in different fields such as social networks, urban and transport planning, economic development, emergency relief, and, recently, public health. In this paper, we briefly describe the state of the art on using mobile phone data for public health, and present the opportunities and challenges that this kind of data presents for public health.

## Introduction

Since 2014, and according to the International Telecommunications Union (1), the number of mobile phone subscriptions exceeds the world’s population. This high level of adoption applies to both developing and developed economies and to nearly all socio-economic statuses. As an example of how fast mobile phone adoption is growing, in 2014 the level of mobile penetration ranged from 90% in developing countries to 128% in developed economies, compared to 79–87% in 2011 (1). In fact, the mobile phone has become the most ubiquitous piece of technology in our recent history. What is interesting and powerful is the fact that mobile phones are connected, leaving a digital trace behind, which can be used to analyze and model human behavior at an individual and aggregate levels. The analysis of these digital traces has already been successfully applied in a variety of fields, including urban planning (2); modeling human mobility (3); understanding social network structure (4) or measuring economic development (5).

In this paper, we outline the immense opportunities that mobile data – as it is captured from the mobile network infrastructure – presents for public health. In particular, Section “Mobile Network Data” presents the different sources and types of mobile network data available and describes their advantages and limitations. Sections “Mobile Network Data” and “Behavior and Public Health” outline how mobile data can be used for public health. Finally Section “Challenges of Using Mobile Data for Public Health” highlights the main technical, regulatory, legal, and ethical challenges that come associated with this opportunity and presents possible strategies to overcome them.

## Mobile Network Data

A mobile (or cellular) network is a wireless network composed of towers, called Base Transceiver Stations (BTS), which give coverage to a geographical area. The coverage area of each individual BTS is called a *cell* and is typically divided in three sectors each one covering 120°. Although this is the typical case it is possible for a BTS to have just one-directional sector or more than three sectors to handle areas with high density of population. The geographical area covered by a BTS depends mainly on the power of the individual antennas. Depending on population density, BTS coverage typically ranges from <1 km^2^, in dense urban areas, to >4 km^2^, in rural areas. For simplicity, it is common in the literature to assume that the cell of each BTS is a 2-dimensional non-overlapping polygon, which is typically approximated using Voronoi diagrams. Simply, this approach gives a good approximation of the coverage area of each BTS. In practice, to build the “real” diagram of coverage, one has to consider several factors in the mobile network, including the power and orientation of each antenna.

**Figure 1:**
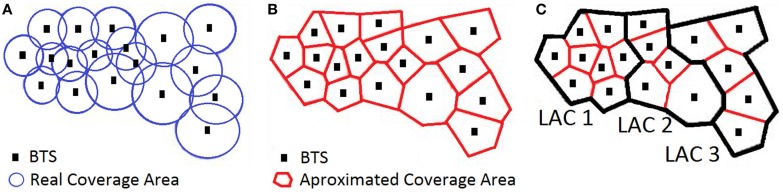
**(A)** Original coverage areas of BTS, **(B)** approximation of coverage areas by Voronoi diagram and **(C)** geographical representation of LACs.

In order to optimize signaling, BTS are grouped in Location Area Networks (LACs), which typically contain multiple BTS, ranging from 10 to more than 100, depending on the communication needs. LACs help determine the current location of a mobile phone within a cellular network without having to go down to the BTS level. Figure 1A depicts a set of BTS with the original coverage for each cell, Figure 1B the simulated coverage obtained using Voronoi diagrams and Figure 1C the grouping of BTS into LACs (3 in the Figure).

When a cell phone is connected to the network, it notifies the BTS where it is located in order to be able to provide communication services to the user. There are two types of notifications which generate two different types of data: (1) *Event-driven* cell phone network data, which refers to information collected when a service (e.g., call, SMS, MMS, Internet access, etc.) is actively requested by the user; and (2) *Network-driven* cell phone network data, which captures periodic location information triggered by updates requested by the network in order to know where the cell phone is located.

### Event-driven cell phone data

Traditionally, event-driven cell phone network data have been referred to as Call Detail Records (CDRs), which store information that is needed for invoicing purposes. The information stored in a CDR is not necessarily standardized, and can vary between different mobile operators. In general, once a mobile subscriber connects to the network and uses a service (such as sending or receiving a call, an SMS, an MMS, etc.) the BTS logs, among other data, the encrypted originating and destination phone numbers, a timestamp, the call duration, and the identifier of the sector and the cell tower that provided the communication to both cell phones. These identifiers give an indication of the geographical location of the mobile phone at that specific moment in time. However, no information about the position of the mobile phone within a cell is known. Additional information, such as error codes, identifiers of the network operators, type of contract, etc. can also be included in the CDR. Also, if the service is a phone call, the CDR typically contains the set of cells used during the conversation if there is a change of area of coverage because the user is on the move, i.e., the handover information between BTS. CDRs have databases for associating sectors to longitude and latitude, and also for identifying network operators, error codes, etc. Note that if the phone is not actively using mobile network services, there will be no information generated in the CDRs.

Table 1 presents an example of CDRs for three calls where handover information has been also collected. It contains the originating encrypted phone number, the destination phone number, the date and time of the phone call, and identifier for the network operator of the originating phone number, and identifier of the network operator of the destination phone number, the duration in seconds, the sector or sectors of the originating phone number while the call took place, the sector or sectors of the destination phone number while the call took place, and a code that indicates if there was an error during the communication. In this example, we assume that CDRs are generated by the network provider identified with 1, which implies that the CDR will only be able to collect sector information if the phone number is in the network of provider 1. The first call is from encrypted numbers 3643533533 to 5643786412 where, the originating phone number belongs to network operator 1 but the destination to network operator 3. This implies that only the sector or sectors for the originating phone number will be available. The second call is between two cell phones of operator 1, and as such the sectors of both phones can be captured. In this case, the destination phone was moving during the call and the list of sectors is given in the corresponding field. The third entry corresponds to a call from encrypted number 5643786412, which is a phone from network operator 3–3643533533. In this case only the sectors for the destination phone are available. Note that because handover information is being collected, the fact that there is only one sector implies that the phone did not change sectors during the duration of the phone call.

**Table 1:** **Exemplary CDRs representing three different calls**.

Originating	Destination	Date/time	Op-orig	Op-dest	Duration	Sector-orig	Sector-destin	Code
3643533533	5643786412	01–01–14/17:22	1	3	56	2354626		0
3643533533	8641278633	01–01–14/19:22	1	1	432	2354626	2354666	0
							2354667	
5643786412	3643533533	01–01–14/19:56	3	1	167		2354626	0

Moreover, when a mobile phone connects to the Internet, the BTS also creates a record of the data connection events. As in the case of CDRs, the content is not necessarily standard, but typically contains an encrypted identifier of the mobile phone, the time and date of the event, information about the website visited, number of bytes transferred, control codes, etc. The logs that contain Internet access information are referred to as *Internet access logs*.

Both CDRs and Internet access logs constitute longitudinal *digital traces* of human behavior from, which we can infer communication patterns, location, social network links, and browsing history. In particular, four types of variables are computed from CDRs and Internet access logs: (1) consumption; (2) social; (3) mobility and (4) personal interests. We refer the reader to Sections “Modeling Mobility from Mobile Network Data” and “Building Behavioral Models from Mobile Network Data” for examples of the variables that are typically computed from these data logs.

This information – anonymized and in many cases aggregated – allows to model human behavior at both individual and aggregate levels. At an individual level, previous work has inferred behavioral changes (6), user demographics (7), credit scores (8), personal characteristics (9) and sleeping patterns. From an aggregated perspective, researchers have been able to characterize the mobility of an entire population (10), transportation flows (11), the exposure to air pollution (12), predict crime (13), and infer socio-economic indicators of regions (5, 14).

### Network-driven cell phone data

A cellular network needs to be aware of where cell phones are located in order to provide services (e.g., routing calls, delivering SMS, etc.). For this purpose, network events are generated to update the location of the cell phones. Network-driven cell phone data, sometimes referred in the literature as passive monitoring (15), is generated by events triggered by the network, even if the user did not necessarily request any services. As a result, there is an entry for each event of the network, with its associated timestamp and the BTS that handled it. As with CDRs, no information about the position of a mobile phone within a cell is known. The set of fields typically stored include: (a) an identifier for the type of event; (b) the encrypted phone number that triggered the event; (c) the identifier of the BTS or set of BTS that handled the event; (d) the date and time of the event and an (e) error code. A network-event database will also contain tables that associate the identifier of the event with the event and the error codes with the description of the errors.

Typically, there are three main types of events that the network captures. Note that different network providers capture all or only a subset of this information, depending on the sensor infrastructure deployed on the network:

- *Changing LACS*: e.g., following the case of Figure 1, if one phone moves from a BTS in LAC1 to a BTS in LAC2 an event will be generated indicating that the mobile phone was connected to a BTS in LAC1 and has moved to a BTS in LAC2.
- *Switching the phone on and off*: in this case, the BTS where the phone was last connected is registered.
- *Periodic location update request (paging)*: if none of the above took place in the last few hours, a location request will be issued which will register the identifier of the BTS and the corresponding LAC that the mobile phone is connected to. The time parameter is typically between 2 and 4 h, i.e., if the network lacks information from the phone for the last 2–4 h, a location request will be issued.

Finally, making or receiving calls or SMS, and the handover between BTS during a call, also generate an update in the network, which is also captured and included as network-driven data. In this case, and contrasting with CDRs, the event only generates the location of the phone and the date and time, with no information about the other side of the interaction (i.e., the other cell phone) or its location. As a result, the location information contained in network-driven datasets is much denser than when only considering CDR information, but cannot be used to derive social variables.

Up to now network-driven data have been used mainly for estimating traffic congestion, raising alerts, and inferring average travel speeds (11, 16, 17).

### Additional network data

In this section, we present additional mobile data that has been used in the literature, namely: (1) simulation of CDRs and/or network-driven data; (2) synthetic generation of CDRs; and (3) signal triangulation of mobile data. The first two can be used when it is not possible to have access to real cell phone network data, while the third one addresses the limitation of location resolution by considering extra information from the network.

#### Simulation of CDRs or Network-Driven Data

This case implies locally capturing with a mobile app the interaction of the cell phone with the network. The identifier of the BTS to which the mobile phone is connected is available to the phone. This information can be locally stored in combination with other information, such as the signal strength, other available BTS, etc. and can be used to simulate the network-driven data that the cell phone network would generate. Moreover, if the mobile app captures interactions – such as calls and SMS – CDRs can be simulated and used for later studies. An example of this approach can be found in Ref. (18).

#### Synthetic CDRs

In this case, synthetic traces are generated from real human behavioral models – typically in the form of calling patterns and/or mobility patterns. There are commercial tools available for such purposes such as the Call Detail Record Generator (19) and the CDR-Generator (20). The technical complexity of these approaches lies in the creation of models that capture real human behavior. The work done by Isaacman et al. (21) presents a synthetic generation tool called WHERE that creates synthetic models by capturing the statistical properties of real CDR traces. The main advantage of this approach is that there are no privacy concerns as the information being used is synthetic and does not correspond with any real mobile phones.

#### Signal Triangulation of Mobile Data

Both CDRs and passive network information cannot determine the location of a mobile phone within a cell. In order to be able to obtain a more refined location of the cell phone within the network additional information needs to be collected, such as the attenuation from the antennas and the strength and the length of travel time of the signal. With that information triangulation techniques can be applied to estimate the distance of a phone from the BTS tower.

### Strengths and limitations of the different types of network data

Each one of the previous types of mobile data has its own technical strengths and limitations that we discuss in this section. Other limitations such as privacy or ownership of the data are discussed in Section “Challenges of Using Mobile Data for Public Health.”

Regarding CDRs, we can identify two inherent limitations: (1) location is captured only when a service takes place, resulting in *low temporal resolution*, and (2) the captured location approximates the actual position, resulting in *coarse spatial granularity*. If Internet access information is also captured in the CDRs, then the temporal granularity limitation is not as strong, because typically the frequency of access to data by users and apps installed on their smartphones is high (e.g., several times per hour). Moreover, a variety of models have been proposed in the literature to estimate location at each moment in time, covering also all the time in between two consecutive entries in the CDRs. Song et al. (10) demonstrated that the location at each moment can be estimated with 93% accuracy, assuming that the phone is used an average of 0.5 times per hour and that an individual visits more than two locations during the acquisition of a training set.

Note that network-driven cell phone data have higher temporal resolution than CDRs, as the information is captured independently of the use of the mobile phone, i.e., the network has information even if a phone is not being used. Nevertheless, it still has coarse spatial granularity. Triangulation techniques are typically carried out to address this limitation. However, it is extremely complex to capture triangulation information for all the mobile phones in a network. Therefore, it is usually applied only to a small sample of phones.

The main strength of network-driven data is the fact that information is captured for all users independently of their actual use of the phone, which implies that mobility models can be created for all mobile phones. Nevertheless, no information regarding the social network of the individual is captured in network-driven data as previously explained.

CDRs do contain the information needed to construct social interactions but the mobility models that can be computed from CDRs are much more limited when compared to those built from network-driven data due to the sparse temporal granularity of CDRs.

In the context of public health, both types of mobile network data provide immense opportunities, in particular in developing economies where the acquisition of public health-related information is costly and limited.

In the remainder of the paper, we discuss the opportunities and challenges to leverage mobile network data for public health. In particular, we focus on the ability to *model mobility* (to e.g., monitor human migrations and target interventions in the case of e.g., epidemics or natural disasters) and to capture *behavioral routines* from this data (e.g., to infer significant behavioral changes and to assess mental health status).

## Mobility and Public Health

The mobility of individuals and entire populations is of paramount importance for public health, particularly in the case of potential pandemics, environmental risks, and natural disasters. Mobility characterization is key to predict the spatial and temporal risk of a human-transmitted infection; to model the spatial spread of drug resistance by pathogens, such as malaria; to understand human migrations after natural disasters or emergency situations; and to quantify exposure to air pollution or other environmental chemicals, with major implications in control and elimination programs in public health (22).

The traditional approach to analyze mobility patterns is based on household surveys and information provided from census data (23). These methods allow for a clear understanding of demographic biases and motivations pertaining to mobility patterns. However, these traditionally collected datasets suffer from recall bias and limitations in the size of the population sample involved in the analysis, mainly due to excessive costs in the acquisition of the data (22). Moreover, survey or census data provide a snapshot of the population dynamics at a given moment in time. However, in the case of public health, it is of paramount importance to obtain a picture of mobility patterns and fluctuations in a continuous manner, particularly during emergencies (such as an outbreak of a potential pandemic or disasters) in order to support decision making or assess the impact of government measures and restrictions to maximize the impact of interventions. In such cases, public health workers typically count people at transportation hubs manually.

The work done by Tizzoni et al. (24) and Wesolowski et al. (22) focused on comparing traditional mobility surveys with the information provided by CDRs specifically to model the spread of diseases. The findings of both papers recommend the use of CDRs, by themselves or in combination with traditional sources, to improve the accuracy of the epidemic situation under study. Moreover, Wesolowski et al. (22) focus the study in low-income settings and developing economies, where the availability of surveys is highly limited, thus highlighting even more the potential of using mobile network data for public health.

### Modeling mobility from mobile network data

Human mobility models derived from mobile network data have the potential to overcome the shortcomings of traditional methods in the context of public health. Given the geolocation (longitude and latitude) of every BTS in the mobile network, CDRs and/or network-event data would enable to infer the approximate locations of mobile phones, which have served as a foundation to develop human mobility models both at an individual and a population level (25). With respect to mobile network data and mobility, there is an important consideration to take into account: its spatial and temporal resolutions as described in Section “Strengths and Limitations of the Different Types of Network Data.”

Typical mobility variables that can be computed from CDRs include the total number of commonly used BTS, the radius of gyration (i.e., the root mean squared distance between the set of BTS’s and their center of masses), the total distance traveled, the diameter of the area of influence (i.e., the geographical area where the user spends his/her time doing daily activities, which is computed as the maximum distance between the set of BTS’s used to make/receive calls), all of them over a specific time period. We direct an interested reader to Ref. (26) for details on human mobility models from mobile network data.

Moreover, recent work has combined mobility and social information, which is of paramount importance for public health, particularly in the context of humanly transmitted infectious diseases. Calabrese et al. (27) and Wu et al. (28) have found that calling people while being connected to the same BTS is a good proxy for face-to-face interactions: people are more likely to physically interact before and after such an event happens. They also discovered that the number of inferred face-to-face meetings decreases as the distance between the homes of the two users increases and were able to predict when and where people would meet. In related work, Farrahi et al. (29) showed that a wide range of contact tracing strategies may significantly reduce the final size of an epidemic, by mainly affecting its peak of incidence.

Selecting a representative population sample and aggregating individually inferred mobility patterns are a first and necessary step when characterizing population mobility dynamics. An important advantage of mining mobile network data for this purpose is the ability to discard the CDRs of the individuals who do not use their phone often enough to generate a meaningful sample (30). Another advantage is that the aggregated nature of the analysis minimizes privacy concerns while is still of great value for public health.

### State of the art

Frías-Martínez et al. (25) proposed an epidemic spread model that captures population’s mobility and social patterns and quantifies the changes of these patterns over time. The analysis of individual mobility patterns was based on computing the location of mobile users at the BTS level and estimating locations at each moment in time. The users’ social networks were modeled by inferring close relations in the communication patterns reflected in the CDRs. The epidemic spread model assumed that two users that belong to the same social network are more likely to be physically close if detected in the vicinity of the same cell tower, which increases the probability of an infection transfer between them. The approach was validated using CDR data collected during the H1N1 outbreak in Mexico in 2009, and it showed that the peak of the infection was reduced by approximately 10% and postponed for approximately 40 h as a result of the government actions.

Similarly, in a series of studies (30–32), the authors analyzed the CDRs of almost 15 million Kenyan mobile subscribers in an effort to understand the transmission of malaria; the approach was grounded in the fact that human mobility significantly contributes to the spatial spread of malaria, even more than mosquito dispersal. Mobile network data collected over the course of 1 year was analyzed to establish the primary locations of individuals (i.e., where they spent the majority of time) and destination and durations of each journey thus building the population mobility model. The mobility patterns were coupled with malaria prevalence data to infer both the residents’ and visitors’ probability to be infected, and ultimately to map the routes of parasite dispersal – regions where the disease originated and where it was transmitted and to locate high-risk spots in order to improve malaria control programs. Tatem et al. (33) and Chuquiyauri et al. (34) also explored the transmission of malaria, though on a smaller scale, and focused on the parasite importation rates from Tanzania to Zanzibar, revealing that a few people account for most of the risk for imported malaria. Le Menach et al. (35) combined cell phone data and ferry traffic between Zanzibar and mainland Tanzania, and concluded that Zanzibar residents traveling to malaria endemic regions were estimated to contribute 1–15 times more imported cases than infected visitors.

Human travel is investigated also in the context of the Dengue virus transmission in Iquitos, Peru (36, 37). The study in Peru was conducted on a small scale, involving 126 individuals, and relying on global positioning system (GPS) to locate the individuals. In addition to exploring the potential of quantifying mobility patterns with respect to the risk of transmission of Dengue in resource-poor settings, the authors also focused on the acceptance of GPS devices in longitudinal studies and identified a number of issues, namely: health effects, care of the units, and privacy and confidentiality of the information (36).

After Haiti’s earthquake in January 2010, followed by a cholera outbreak in October 2010, researchers at the Karolinska Institute in Sweden analyzed daily movement data from two million mobile phones and were able to: (1) identify critical areas of the cholera outbreak (38), and (2) quantify the population that was affected by the disaster and their movements in the following period (39). This study illustrated the tremendous value for public health and emergency services officials of mobile network data when made available right after a disaster takes place.

In 2014, we faced the worst Ebola outbreak in our history. Given the relevant previous work and the ubiquity of mobile phones, a small group of researchers – including ourselves – and Big Data experts advocated the use of aggregated and anonymized CDRs to help fight against the disease. However and despite these efforts (http://techcrunch.com/2014/11/08/using-big-data-to-fight-pandemics/), we did not succeed, mainly due to regulatory and legal limitations, combined with possible lack of incentives, potential unintended consequences (e.g., the affected areas are areas with current or recent civil unrest) and lack of technical expertise (40).

In addition to understanding population mobility in case of epidemics or natural disasters, mining mobile network data can provide valuable information for ongoing routine public health surveillance (i.e., regardless of the crisis outbreak). One such example is the analysis of individual exposure to air pollution and the implications for public health impact assessments (12). Liu et al. (12) have proposed to evaluate the impact of traffic-related air pollution on public health by analyzing individual trajectories assigned to both people and vehicles. The model takes into account the vehicle type, speed, and emission rates. Although the study was not based on empirical evidence, the authors argue that this approach could help identify trajectory patterns of particularly exposed groups of individuals, and allow for new perspectives in public health research.

Orange has launched two public challenges to the research community where they have shared aggregated and anonymized CDRs from Ivory Coast and Senegal in the D4D challenges (41). While not focused exclusively in public health, there are a number of interesting scientific papers from the D4D challenges that use CDRs for the containment of epidemics, such as the work by Lima et al. (42), that shows that information campaigns are more effective in limiting the epidemic than quarantine measures; and the work by Kafski et al. (43) according to which messages recommending people not to cross into other communities, even if only followed by a fraction of the population, can have a big impact on the spread of an epidemic.

## Behavior and Public Health

Individual and aggregated human mobility is certainly a key variable to measure, model and predict in public health. As we have seen in the previous section, human mobility models can be built from passively collected mobile network data, with great promise to help decision making in public health, particularly when fighting against an infectious disease, facing the risk of a pandemic or when dealing with the consequences of a natural disaster.

However, mobility is not the only human characteristic that can be inferred from mobile data. As previously seen, consumption patterns and social variables can be inferred from CDRs and Internet logs, enabling the construction of rich models of human characteristics and behavior. One area of public health where we believe this kind of data could have significant impact is mental health, for which monitoring behavior becomes central in the treatment and management of mental disorders (44).

Mental health problems account for 20% of the disease burden worldwide; one out of four individuals suffers mental health problems in a given year (45), it is the third most common reason to visit a health center (46), in addition, suicide – with a yearly rate of 800,000 worldwide – is recognized to be a major public health issue (47). Though mental health has long remained outside the public health practice (48), it has been receiving an increasing level of attention in public health action plans that suggest the strategy to be shaped around its prevention (49). However, the traditional model of episodic care is suboptimal to prevent mental health outcomes and improve chronic disease outcomes (50–52).

In order to assess human behavior in the context of mental wellbeing, the standard clinical practice relies on periodic self-reports that suffer from several shortcomings, including memory dependence, recall bias, subjectivity, and influence of the current mood of an individual. Besides, individuals with mental conditions typically visit doctors when the crisis has already happened or is underway thus reporting limited information about precursors and making it impossible to eventually prevent the crisis onset. The challenge of diagnosing a crisis or a disorder is further exacerbated in low and middle income countries where 75–85% of patients with severe mental disorders are unable to access appropriate health care services and to receive treatment (53).

Thanks to the ubiquity of mobile devices, today, we have the ability to monitor human behavior outside of clinical settings and without having to depend on self-reported information. The opportunity to passively collect large-scale human behavioral data is key to diagnose early and prevent mental conditions, mitigating the pressure on healthcare systems, and ultimately bringing important benefits for public health. One of the main functions of public health is the assessment and monitoring of the health of communities at risk to identify health problems and priorities, the promotion of health and the delivery of disease prevention services (48). Human behavior monitoring and understanding is a key enabler of these functions.

### Building behavioral models from mobile network data

The role of mobile technology in healthcare has been recently emphasized for its opportunity to extend health interventions beyond the reach of traditional care – the approach referred to as Mobile Health (mHealth) (54, 55). Mobile phones can have a significant impact on mental healthcare through sensing, analyzing, and affecting human behavior (56), enabling the development of mental health prevention, promotion, and management tools. The ultimate goal would be to move some of the mental healthcare tasks to daily life outside of clinical settings. Although mHealth applications have shown the potential to overcome the limitations of self-reporting methods, the widespread adoption of mobile health applications is still limited due to (a) a lack of historical information about a patient before installing the application, (b) considerable consumption of phone resources by the application (e.g., battery, CPU, memory), (c) limited reach – as only 1 out of 5 persons worldwide owns a smartphone required for installing mobile health applications (57), and (d) lack of portability (e.g., app requiring a specific mobile OS). In this regard, passively collected mobile network data can overcome the drawbacks of mobile phone applications while still serving as an accurate proxy of human behavior (58).

As previously described, both CDRs and Internet access logs constitute longitudinal *digital traces* of human behavior from which we can infer communication patterns, location, social network links, and browsing history. Different kinds of variables can be computed from CDRs and Internet access logs, including:

(1) *Consumption* variables, such as the total number of incoming and outgoing calls received by a user; the average duration of incoming and outgoing calls; the total expenses in phone calls; the total number of incoming/outgoing SMS; the ratio of incoming/outgoing SMS versus all communications; the amount of data transferred and received; the amount of time spent on the Internet, all of them over a specific time period (e.g., day, week, month.);
(2) *Social* variables, such as the in and out degree of the user’s social network, built from the call graph or the graph created from the CDRs, and the centrality and total degree of the network;
(3) *Mobility* variables, such as the total number of commonly used BTS, the radius of gyration (i.e., the root mean squared distance between the set of BTS’s and their center of masses), the total distance traveled, the diameter of the area of influence (i.e., the geographical area where the user spends his/her time doing daily activities, which is computed as the maximum distance between the set of BTS’s used to make/receive calls), all of them over a specific time period; and
(4) *Personal interests* variables, such as the topics or categories of the most accessed Web services and mobile apps over a specific time period.

In addition to the variables above, other aspects of human behavior can be inferred from these logs, such as sleep patterns (obtained from the timestamps of the last/first entries in a day) and commuting routines and distances (obtained after inferring the user’s home and work locations).

From these variables, we can build models of individual and aggregated human behavior that are relevant for mental health conditions, particularly to analyze aspects of daily routine and lifestyle that may be valuable to (a) monitor the condition, and (b) detect behavioral deviations that are indicative of a crisis (44).

From the perspective of public health, mining mobile network data can potentially enable us to identify populations and situations in which an intervention (such as a message, a phone call or a visit) can trigger positive behavioral change or encourage adherence to the therapy, which would contribute to improving public health and lower healthcare costs. In a recent report, the World Health Organization has emphasized the role of public health interventions to improve mental health (51) and in this respect mobile network data would enable to develop tools to support such public health actions.

### State of the art

Mobile applications have been proposed for symptom assessment, psycho-education, resource location, and tracking of treatment progress (55). To the best of our knowledge, there have neither been attempts in research nor in commercial services to leverage mobile network data for monitoring patients with mental conditions and for identifying groups at a particular risk. This section provides an overview of related studies that rely on smartphones to either monitor behavior in the context of mental wellbeing or to deliver interventions.

#### Monitoring Human Behavior with Mobile Phones

The behavioral data collected through mobile phones has been exploited to recognize mood (59) and stress (60), to understand triggers of mood changes (61), and to help manage stress, anxiety, and mood disturbances (55). Specifically related to mental disorders, the EU FP7 project MONARCA (62) investigated the feasibility of providing a smartphone-based platform to continuously acquire behavioral data of bipolar disorder patients to detect significant changes in their behavior related to maniac and depressive episodes. Gruenerbl et al. (63) demonstrated that a smartphone can be used as a “measurement device” for supporting bipolar disorder patients, achieving high accuracy both in recognizing the current state and in predicting state change. In a similar line, there are a few recent off-the-shelf mobile applications, such as Ginger.io and Mobilyze, that aim to signal changes in one’s behavior (e.g., staying at home for several days) and present the inferred behavioral parameters to the specialists.

#### Delivering Interventions with Mobile Phones

Mobile phones have been increasingly emphasized as a platform that can be suitable for delivering feedback and providing behavioral therapy thanks to the fact that people habitually carry mobile phones and that they are able to unobtrusively sense and analyze human behavior. Lathia et al. (56) proposed a system to provide large-scale behavior change interventions based on sensing a specific set of user’s activities, learning behavioral models, and delivering tailored behavioral change interventions at a suitable time. In particular, mobile applications that target mental health have demonstrated a high potential to be effective in providing interventions and improving treatment accessibility. The current literature reports several recent studies that have designed mobile intervention approaches to improve mental health conditions, including depression (64, 65), anxiety (66), stress (67), bipolar disorder, and schizophrenia (68). Most of these studies involved a small number of participants thus have limited scientific evidence about the efficacy of the interventions (specifically in the long term) (69, 70). However, considering these promising preliminary results and an increasing number of studies that aim to validate mobile-phone based interventions, they illustrate the potential to move toward preventative models, which can be of a particular benefit for public health (49).

When it comes to non-smartphone-based interventions, user-interaction possibilities become limited to the available channels (such as SMS or calls) as opposed to interactive interfaces in smartphone applications. Nevertheless, texting was been shown to be a simple but powerful way to achieve positive behavioral changes: Fogg et al. (71) presented several health domains in which SMS-based interventions could be effective and they highlighted specific use-cases of using text messages to educate or notify people, collect user data – such as answers to specific questions or self reports – and connect individuals and groups.

Given the lack of prior work on using passively collected mobile network data for mental health, we believe there is a tremendous opportunity to have positive impact in this domain. However, such an impact will only be achieved when technical, regulatory, legal and ethical challenges are addressed, as described below.

## Challenges of Using Mobile Data for Public Health

Using mobile network data for public health applications implies a series of challenges, not only technical but also regarding privacy, security, regulation, and legislation.

### Privacy, regulation and data security

Despite the fact that mobile network data can provide groundbreaking opportunities for public health, taking advantage of this data in practice is by no means trivial. Storing, accessing and processing data that contains personal sensitive information, such as location and mobility, Internet logs, call and messaging patterns, as well as information related an individual’s social network must adhere to data privacy laws and a clear ethical code of conduct. Even if the data is encrypted and it is processed with full informed consent from all users, there is still a risk of deducing identities from the data, particularly when combined with other data sources. Thus, ownership, transparency and control of personal data are important topics that would need to be addressed (72).

#### Deducing Identities from Anonymized Individual Data

Despite the algorithmic advancements in anonymizing data and hashing identifiers, it has been shown that it is feasible to deduce identities from anonymized human behavioral data, particularly when combined with data from different sources. For example, Zang et al. (73) have shown that if home and work addresses were available for some users, up to 35% of users of the network could be de-identified just using the two most visited towers (which will probably be home and work). Taking this idea further, de Montjoye et al. (74) have demonstrated how unique the mobility information is for each individual and how that information can be used to de-identify users with an accuracy of 95%.

As a consequence, the vulnerability of the mobile network data to malicious attacks represents one of the obstacles for granting access to this data to researchers for human behavior modeling. Nevertheless, the issue of deductive disclosure is a common problem with a wide variety of personal datasets. As argued by Eagle (72), data sharing protocols must be developed that could be similar to those long-used by the medical community. In addition, several privacy-enhancing technologies for mobility data have been proposed by the scientific community (75). Two solutions described by Krumm (76) are *location obfuscation* (77), which consists of slightly altering location information in irreversible ways such that it does not reflect the real location but is still representative of the phenomenon under study; and *k-anonymity for trajectories* (78), which ensures that individual trajectories can only be released if there are at least k-1 trajectories that are indistinguishable from the specific trajectory to be shared and analyzed.

An additional area of research consists of understanding the maximum levels of individual, spatial and temporal aggregation that would still enable to make accurate models and inferences while maximizing privacy preservation thanks to the aggregation. We leave this topic for future work.

#### Data Ownership

Mobile network data is generated by mobile subscribers as digital traces that are collected as a consequence of their longitudinal use of mobile network services. This raises questions that do not have clear-cut answers about who owns the data and who can control its usage. Even when the data is analyzed on an aggregated level, the individuals are typically unable to opt out from the data analysis and to remove their data from the aggregated datasets (72). This issue requires legislation *per se*, and the lack of international standards in this area can result in public distrust in using this technology. Yet, the examples of storing and accessing extremely personal data about human behavior can be found in various domains such as banking, healthcare, education, social networks, and other online services, without a commitment to deliver the outputs for public good.

There is a need for updated technical standards, regulation and legislation before we can leverage this new type of human behavioral data for public health. To address privacy concerns, privacy-preserving technical solutions need to be adopted and users need to be given full control over which data they feel comfortable sharing for social good (72). As previously described in Section “Additional Network Data,” synthetic CDR data generation could be an alternate approach to address privacy and data ownership constraints while still adding value (79).

There are several successful examples of sharing aggregated and anonymized mobile operator logs to the research community such as Telefonica’s Datathon for Social Good in UK (80), Orange’s D4D challenges (41), Telecom Italia Data challenge (81), and Digicel’s data access to researchers after Haiti’s earthquake in 2011 (39). These examples illustrate how obstacles related to privacy and data security can be resolved in certain cases. Such single-case agreements should evolve into a set of official protocols that would expedite the process and also minimize the risks in providing access to this data (privacy related and non-intended consequences) (72).

In case of epidemics or natural disasters that would require prompt access to mobile network data, having regulation and legislation on the use of anonymized and aggregated mobile data, together with technical expertise, shared best practices and a clear code of conduct with dealing with mobile data would enable an appropriate response in near real-time when needed (40, 82).

### Social considerations

Despite the wide adoption of mobile phones worldwide, there are still hundreds of millions of people who do not have a mobile phone or who make a very limited use of their devices. These people are at risk of being excluded by any mobile data-driven analyses. Researchers, decision makers and health officials working and making decisions based on the analysis of these large-scale datasets should certainly keep this factor in mind. Moreover, as we change to a data-driven society where our decisions are increasingly based on the results of data analytics, we need to make a conscious effort to avoid creating a digital divide between those who have access to data and those who do not; or those who have the expertise and knowledge to analyze this data and make sense of it, and those who do not.

Finally, as it has been evidenced by some of the proposals presented to the D4D challenge (41, 83) it is crucial to take into consideration potential unintended consequences that could arise from the public release of the insights gathered from the analysis of this kind of data even if it is for public health purposes. For example, the inference of migration patterns to better understand and anticipate the spread of a pandemic could put certain populations at risk in areas with civil unrest or conflict between different ethnic groups.

### Technical and research challenges

Mobile network data offers tremendous advantages such as near-universal access and passive collection without the need for human intervention. However, it is also subject to limitations in data collection (e.g., gaps in location tracking, no access to the phone sensors such as accelerometers, light sensor, GPS, etc.) and in user-interaction (e.g., instead of interactive interfaces provided by an app or an online dashboard the interaction is reduced to mobile network communication channels such as SMS messages or calls). Therefore, further research is needed to provide a deeper understanding of the values and limitations of mobile network data for public health.

While the potential and the promise to have positive impact are large, there are significant technical and research challenges that would need to be addressed before we can fully leverage this data for public health, including:

#### Scientific Validation, Lack of Ground-Truth

The majority of mobile health applications are not scientifically validated thus cannot be officially included in the standard healthcare practice (70). Similarly, conducting further research and empirical validation of the approaches that rely on mobile network data is a critical step toward the adoption of mobile network data based approaches in public health practice. A prerequisite is having reliable ground-truth that is often a non-trivial task.

For example, in the case of mobility the difficulty in obtaining reliable ground-truth relates to the previously discussed shortcomings of surveys and census methods, including limited-size of population samples, obsolete data and recall bias. Wesolowski et al. (22) reported the discrepancy between the mobility reported through surveys and the one modeled through the CDRs analysis in Kenya, with the significantly lower volume captured by the former method. As possible reasons, the authors speculated about the fact that working age men are often absent during community hours, that the details about trips taken may be forgotten or not accurately reported and that it was challenging to conduct the survey on a large scale. Conversely, the sample of mobile phone subscribers could be biased toward more educated urban males (note that for privacy issues no demographic data about mobile subscribers was available) and also due to phone sharing and multiple SIM card ownership. In such cases, it becomes difficult to assess each of the methods as there isn’t an ultimate ground-truth. However combining the two data sets can give a more complete picture of human mobility than each of the methods can provide independently.

In terms of behavioral changes that are due to mental health conditions, the collection of ground-truth is currently limited to medical records (hospitalizations, doctor’s visits), which in many cases contain a lot of self-reported information. Diagnosing affective disorders, the patients’ episodes and current state is mostly based on both self-reports of behavior and mood and on direct observations by psychiatrists or informal caregivers, which suffers from subjectivity and human errors. Therefore, reliable ground-truth when it comes to mental health is also a challenge as there are neither firm biomarkers nor imaging techniques that reliably diagnose mental conditions (84).

#### Need for Interventions

A promising approach in the use of mobile data for public health would combine the detection of unusual patterns of behavior with interventions delivered directly to the population through mobile phones, specific government measures or their doctors or caregivers (in the case of mental health, for example). Only when we will be able to close the loop between the insights extracted from the data and the humans that generated such data we will be able to gather quantitative evidence of the value of the analyses in the real world and realize the full potential of this technology.

#### Temporal and Spatial Granularity

As previously described, data captured by the mobile network infrastructure has temporal and spatial limitations that need to be taken into account in the analyses. Spatial limitations imply that it is in general extremely difficult to assign a precise physical location to the user from the information contained in the mobile network. Temporal limitations imply that there is only a partial view of the user’s activity, i.e., we can have no information of a user for prolonged time periods. Therefore, it is of paramount importance to provide results that take into account this uncertainty (85) and to develop algorithms that try to overcome the limitations in temporal and spatial granularity.

#### Biases in the Data. Generalization Ability

The tremendous potential of mining big data are undeniable, however the quantity of data does not guarantee the reliability and validity of the approach. Note that the vast majority of large-scale human behavioral data used today is used in an opportunistic manner as the data was originally collected for other purposes (86). A well-known example of this limitation in Big Data Analysis is the recent bias reported in Google Flu Trends when compared to the estimations carried out by the Center for Disease Control. According to Lazer et al. (86) one of the causes of these deviations is the dynamic nature of the algorithms that underlie Google search. However, while Internet services (such as Google, Facebook, Twitter) frequently change (thus making the logs not consistent overtime for e.g., the flu prevalence analysis), mobile network logs are more stable and their generation does not rely on algorithms that change overtime. Yet, mobile data still hides potential risks of overfitting to a small portion of cases for which the biased nature of mobile phone ownership is one of the potential contributors to consider. Wesolowski et al. reported (32) a limited impact of the mobile phone ownership disproportion (toward urban educated males) on population mobility inference. Hence, the selection of the mobile data sample and the validation of the results should be carried out with caution as mobile phone ownership could be skewed due to socio-economic, cultural and demographic factors (87).

#### Real-Time Analysis

Certain public health scenarios, such the risk of a pandemic, require real-time decision making. Being able to access and analyze mobile data in real-time is still a challenge in most countries. Mobile network data is typically collected throughout a specific time period (e.g., one day) and only then pushed to databases such that it is not available in real-time. Data analytics algorithms for streaming data would be needed to be able to process real-time mobile data (88).

#### Combination with Other Data Sources

As we have described in this paper, mobile network data enables us to characterize human behavioral variables that are of paramount importance to public health, such as mobility routines, consumption patterns and social network characteristics. However, for many public health scenarios, it is necessary to combine these variables with variables coming from external data sources, such as public health information or medical records. The linkage of these different datasets poses both technical and privacy challenges that would need to be addressed.

## Conclusion

In this paper, we have described the potential of using different types of mobile network data for public health. In particular, we have focused on the opportunity to model individual and population mobility and to characterize human behavior. The analysis of individual and population mobility patterns in a more objective way and with finer spatio-temporal resolution in comparison to traditional methods opens a door to revolutionize public health. Furthermore, mobile network data can also provide a continuous insight into human behavior that can support the assessment and monitoring of the health of specific communities at a risk, e.g., mental conditions, thus paving the way toward improved health promotion and prevention.

Since the acquisition of mobile network data was not purposefully designed for scientific research and to support public health, its analysis entails technical, legal and regulatory challenges that could limit a practical implementation, including privacy and ethics, potential sample biases, limited temporal and spatial granularity, and real-time analysis.

In order to expedite the adoption of mobile data for public health, a global coordination is needed that would support an efficient dissemination of best practices, establish and update existing regulation and legislation and improve technical standards.

In our vision, the ultimate goal would be to complement traditional approaches and to enable the shift from population-based and reactive healthcare to personalized, proactive, and preventive healthcare.

## Conflict of Interest Statement

The authors declare that the research was conducted in the absence of any commercial or financial relationships that could be construed as a potential conflict of interest.
